# Standard Diffusion-weighted MRI for the Diagnosis of Central Retinal Artery Occlusion: A Case-Control Study

**DOI:** 10.1007/s00062-020-00955-6

**Published:** 2020-09-16

**Authors:** L. A. Danyel, G. Bohner, F. Connolly, E. Siebert

**Affiliations:** 1 Dept. of Neurology, University Hospital Charité, Augustenburger Platz 1, 13353 Berlin, Germany; 2 Institute of Neuroradiology, University Hospital Charité, Berlin, Germany

**Keywords:** Cerebrovascular disease, Stroke, Ischemia, Magnetic resonance imaging, Retinopathy, Blindness, Embolism

## Abstract

**Purpose:**

To evaluate diffusion abnormalities of the retina and optic nerve in patients with central retinal artery occlusion (CRAO) using standard stroke diffusion-weighted magnetic resonance imaging (DWI).

**Methods:**

In this case-control study, DWI scans of patients with nonarteritic CRAO were retrospectively assessed for acute ischemia of the retina and optic nerve. Two neuroradiologists, blinded for patient diagnosis, randomly evaluated DWI of CRAO patients and controls (a collective of stroke and transient ischemic attack [TIA] patients) for restrictions of the retina and optic nerve. We calculated statistical quality criteria and analyzed inter-rater reliability using unweighted Kappa statistics.

**Results:**

20 CRAO patients (60,6 ± 17 years) and 20 controls (60,7 ± 17 years) were included in the study. Sensitivity, specificity, positive and negative predictive values for retinal DWI restrictions were 75%/80%/79%/76% (reader 1) and 75%/100%/100%/80% (reader 2), respectively. Unweighted Kappa was κ = 0,70 (95% CI 0,48‑0,92), indicating “substantial” interrater reliability. In comparison, sensitivity, specificity, PPV and NPV (positive and negative predictive values) for restrictions of the optic nerve in CRAO were 55%/70%/65%/61% (reader 1) and 25%/100%/100%/57% (reader 2). Inter-rater reliability was “fair” with unweighted Kappa κ = 0,32 (95% CI 0,09‑0,56).

**Conclusions:**

Retinal diffusion restrictions were present in a majority of CRAO patients and detectable with reasonable sensitivity, high specificity and substantial inter-rater reliability. Further studies are necessary to study time dependency of retinal diffusion restrictions, improve image quality and investigate the reliability of retinal DWI to discern CRAO from other causes of acute loss of vision.

**Electronic supplementary material:**

The online version of this article (10.1007/s00062-020-00955-6) contains supplementary material, which is available to authorized users.

## Introduction

Nonarteritic central retinal artery occlusion (NA-CRAO) is a medical emergency characterized by a sudden onset of painless, monocular amaurosis due to retinal ischemia. Diagnosis of CRAO is commonly established through clinical and ophthalmologic examination whereby patients present with decreased visual acuity and impairment of visual field, mostly central scotoma [1]. Frequent ophthalmoscopic findings include retinal edema, attenuation or segmentation of retinal arterioles and visible retinal artery emboli [2]. Similar to ischemic stroke, CRAO is mainly caused by emboli originating from large artery atherosclerosis [3–5] or cardioembolism [6, 7], although a wider etiologic spectrum needs to be considered [8–14].

As the risk of recurrent cardiovascular events, including stroke, is elevated and dependent on CRAO etiology, a thorough diagnostic work-up is required [15–18]. Hence, brain magnetic resonance imaging (MRI) is increasingly performed in CRAO patients. So far, MRI studies have focused solely on the detection of concomitant brain infarction or leukoaraiosis [19–22]. Acute ischemic lesions are found in approximately 25% of CRAO patients [19, 21, 22]. Reports of diffusion-weighted imaging (DWI)-related abnormalities of the optic nerve in CRAO, however, have been limited to a few cases [23–25]. To the best of our knowledge, no systematic MRI studies related to distinctive retinal features in CRAO have been published [26, 27].

Hence, the goal of our study was to improve the characterization of typical retinal and optic nerve changes in DWI and determine their diagnostic accuracy for the detection of CRAO.

## Methods

### Patients

In this retrospective case-control study, we evaluated diffusion-weighted MRI scans in patients with nonarteritic CRAO treated at the University Hospital Charité in Berlin, Germany between June 2015 and May 2019. All subjects originally appertained to a prospective CRAO cohort receiving ultrasonographic assessment in our neurovascular department. Patients received ophthalmological and neurological examinations upon admission to our emergency department. Diagnosis of CRAO was based on clinical presentation of sudden, painless and persistent monocular visual loss as well as the presence of ipsilateral relative afferent pupillary defect and characteristic ophthalmoscopic findings. Fundoscopic features indicating ischemia included retinal edema with cherry-red spot at the macula, narrowing and segmentation of retinal arterioles and/or visible retinal artery emboli [28]. Optical coherence tomography and fluorescein angiography were additionally performed to support the diagnosis in patients with uncertain fundoscopic features.

We reviewed demographic data and medical histories (including hypertension, hypercholesterolemia, diabetes mellitus, atrial fibrillation and smoking habits), visual acuities, possible intravenous alteplase therapy and laboratory findings (including lipid profiles and glycated hemoglobin). Etiologic classification of CRAO was based on the TOAST subtype classification system [29] incorporating results from ancillary procedures performed for diagnostic work-up of CRAO patients: cerebral MRI including 3D time-of-flight magnetic resonance angiography (TOF-MRA), craniocervical computed tomography angiography, electrocardiographic monitoring, transthoracic or transesophageal echocardiography. In addition, sonography of extracranial and intracranial arteries, as well as the orbit was performed in all patients.

Lastly, we randomly selected age and sex-matched patients treated for cerebral ischemia (ischemic stroke or transient ischemic attack, TIA) in our neurovascular department between June and December 2017 to serve as our control cohort. All stroke and TIA patients received cerebral MRI scans utilizing the same technical protocol used for CRAO patients and comparable diagnostic work-up.

### Diffusion-weighted MR Imaging Analysis

The MR imaging (1.5 T MAGNETOM Aera or 3 T MAGNETOM Skyra, both Siemens, Erlangen, Germany) was performed as part of routine clinical work-up for patients with CRAO or cerebral ischemia and included routine axial echo planar imaging (EPI-)DWI sequences. DWI b = 1000 s/mm^2^ images were evaluated separately by two board-certified consultant neuroradiologists (both >10 years of experience in MR stroke imaging) who were blinded for patient diagnosis. During the assessment, imaging studies of CRAO cases and controls were randomly presented from a mixed data set. The DWI was considered positive for optic nerve and/or retinal ischemia if a distinct unilateral increase in signal was present in the evaluable parts of the optic nerve and/or the retina, with resultant asymmetry compared to the fellow eye. Qualitative patterns of imaging abnormalities are described. The adapted Fazekas score was evaluated as described previously to quantify leukoaraiosis [30]. Extensive methodological information is given in the online data supplement.

### Statistical Analysis

We used IBM SPSS Statistics (IBM SPSS Statistics for Windows, Version 25.0. IBM, Armonk, NY, USA) to compare clinical and radiological data between CRAO patients and controls through χ^2^-statistics. A *p*-value <0.05 was considered statistically significant. Additionally, standard test quality criteria of retinal and optic nerve DWI in CRAO (sensitivity, specificity, positive and negative predictive values) were individually assessed for each reader (neuroradiologist 1 and 2).

Interrater agreement was analyzed through unweighted kappa statistics using a JavaScript-based online tool from www.vassarstats.net (author: Richard Lowry, PhD, Professor of Psychology Emeritus, Vassar College Poughkeepsie, NY, USA). Observed percentage of agreement Pr(a) and expected percentage of agreement Pr(e) were used to calculate unweighted kappa(κ). The interpretation of agreement for κ was categorized as follows: poor (κ < 0.00), slight (0.00 ≤ κ ≤ 0.20), fair (0.21 ≤ κ ≤ 0.40), moderate (0.41 ≤ κ ≤ 0.60), substantial (0.61 ≤ κ ≤ 0.80), almost perfect (κ > 0.80).

Study approval was obtained from the local ethics committee (Charité, Universitätsmedizin Berlin, EA1/177/19).

## Results

### Clinical Characteristics

**Fig. 1:**
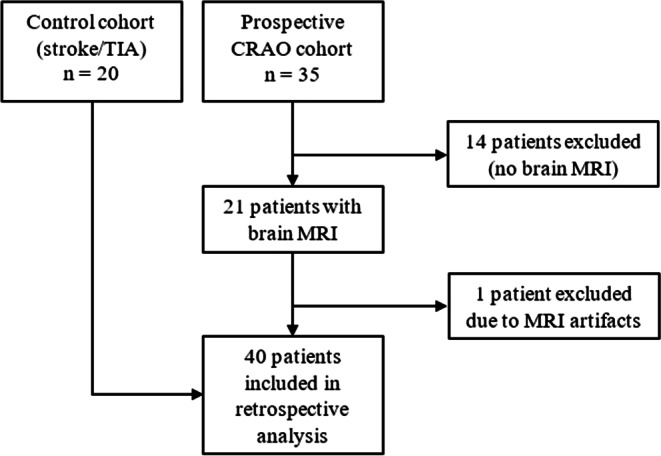
Flow diagram of patient selection process (*CRAO* central retinal artery occlusion, *MRI* magnetic resonance imaging, *TIA* transient ischemic attack)

We retrospectively identified 21 patients who underwent diffusion-weighted MRI. One patient had to be excluded due to severe magnetic susceptibility artifacts secondary to maxillary osteosynthetic material, which rendered assessment of the affected optic nerve and retina impossible. Consequently, the CRAO collective included 20 patients (11 male, 9 female) with a mean age of 60.6 ± 17 years. The controls were 20 age-matched and sex-matched patients treated for cerebral ischemia in our neurovascular department (9 TIA and 11 stroke patients; 11 male, 9 female; mean age 60.7 ± 17 years). Fig. 1 details the patient selection process.

All CRAO patients had Snellen visual acuity of the affected eye equal to or worse than 20/200. In 13 patients (65%) a visible embolus was identified through fundoscopy (visible retinal artery emboli) or sonography of the orbit (visible spot sign [31]). Diagnostic work-up of CRAO patients revealed cardioembolic etiology in 9 (45%) and large-artery atherosclerosis as the source of CRAO in 4 (20%) subjects. In 6 patients (30%), CRAO etiology remained undetermined (no determined etiology or two or more potential causes). In one patient, who did not undergo an echocardiographic assessment and remained without discernible cause of CRAO, the etiologic subtype evaluation was classified as incomplete.

Comparative statistics of cardiovascular risk factors revealed no significant differences in the prevalence of hypertension, type 2 diabetes, smoking and atrial fibrillation between CRAO patients and controls (Table 1). Dyslipidemia was significantly more common in the control group (90% of controls; 60% of CRAO patients; *p* = 0.028). Although recombinant tissue plasminogen activator (rtPA) treatment was more common within the CRAO cohort (7 patients, 35% vs. 3 controls, 15%), the difference did not reach statistical significance (*p* = 0.144).

**Table 1:** Comparative statistics of cardiovascular risk factors, thrombolytic therapy and selected radiological features using χ^2^-statistics

Variables	Control cohort (*n* = 20: 11 stroke, 9 TIA)	CRAO patients (*n* = 20)	*P* Value
*Medical history*			
Atrial fibrillation, *n* (%)	4 (20)	2 (10)	0.376
Diabetes mellitus Type 2, *n* (%)	2 (10)	2 (10)	1
Dyslipidemia, *n* (%)	18 (90)	12 (60)	0.028
Hypertension, *n* (%)	9 (45)	13 (65)	0.204
Smoking, *n* (%)	13 (65)	8 (40)	0.113
rtPA treatment	3 (15)	7 (35)	0.144
*Radiological features*			
MRI field strength 3 T, *n* (%)	3 (15)	5 (25)	0.429
Acute brain infarction, *n* (%)	10 (50)	4 (20)	0.047
Chronic brain infarction, *n* (%)	10 (50)	10 (50)	1

*CRAO* central retinal artery occlusion, *MRI* magnetic resonance imaging, *rtPA* recombinant tissue plasminogen activator, *TIA* transient ischemic attack

### MR Imaging Features

Radiological characteristics of individual CRAO patients are shown in Table 2. In general, MRI in CRAO patients was performed between 17h and 105 h after symptom onset (mean ± SD: 56 h ± 25 h, only patients with definite CRAO onset included; *n* = 16). Field strength of MRI used was 1.5 T for 15 (75%) and 3 T for 5 (25%) of the CRAO patients. The rate of 3 T MRI performed did not significantly differ between CRAO patients and controls (*p* = 0.429; Table 1). The DWI revealed acute cerebral ischemic infarction concomitant to CRAO in 4 subjects (20%), all of which remained asymptomatic. While acute DWI-positive cerebral infarction was significantly more frequent in the control group (50% of controls; *p* = 0.047), the prevalence of chronic brain infarction did not differ between groups (both 50%, Table 1). Leukoaraiosis in CRAO patients was graded as absent in 5 (25%), mild in 10 (50%) and moderate in 5 cases (25%; Fazekas scores 0, 1 and 2, respectively), whereas in control patients it was rated absent in 7 (35%), mild in 9 (45%), moderate in 2 and severe in 2 cases (10% each; Fazekas scores 0, 1, 2 and 3, respectively). There was no significant difference between the CRAO and control group (*p* = 0.299).

**Table 2:** Radiological characteristics of CRAO patients

No.	Age (years)	Sex	CRAO side	MRI field strength (T)	Onset to DWI (h)	Retinal DWI		Optic nerve DWI	
						Reader 1	Reader 2	Reader 1	Reader 2
1	63	f	Right	1.5	67	+	+	+	+
2	48	f	Right	1.5	86	−	−	−	−
3	70	f	Left	1.5	104	+	+	+	−
4	55	f	Left	3	68	+	+	+	−
5	33	f	Left	1.5	59	−	−	−	−
6	46	m	Left	1.5	54	−	−	−	−
7	71	m	Left	1.5	23	−	+	+	−
8	69	f	Left	1.5	90	+	+	+	+
9	68	m	Left	1.5	50	+	+	−	−
10	54	m	Right	3	73	+	+	−	−
11	56	m	Right	3	43	+	+	+	−
12	49	m	Left	1.5	47	+	+	+	+
13	83	m	Right	3	65	+	+	−	−
14	58	m	Left	3	–	+	+	−	−
15	92	f	Right	1.5	–	+	+	+	+
16	26	m	Left	1.5	17	+	+	+	−
17	83	f	Left	1.5	31	+	+	+	+
18	66	f	Left	1.5	–	+	+	−	−
19	79	m	Left	1.5	24	+	−	+	−
20	42	m	Right	1.5	–	−	−	−	−

*CRAO* central retinal artery occlusion, *DWI* diffusion-weighted imaging, *MRI* magnetic resonance imaging

Standard test quality criteria and corresponding confidence intervals of retinal and optic nerve DWI in CRAO are shown in Table 3. Both readers identified retinal DWI restrictions in 15 (75%) of the CRAO patients. Reader 1 falsely attributed retinal diffusion restrictions to 4 control patients (20%) and to the non-affected eye of 1 CRAO patient (rated false negative for the affected eye). The assessment of reader 2 did not yield any false positive results. Sensitivity and specificity rates for retinal DWI restrictions were 75%/80% (reader 1) and 75%/100% (reader 2). Positive and negative predictive values (PPV/NPV) were 79%/76% (reader 1) and 100%/80% (reader 2). Unweighted κ for retinal DWI restriction in CRAO was κ = 0.70 (95% CI 0.48–0.92), indicating substantial interrater reliability.

**Table 3:** Standard test quality criteria for retinal and optic nerve diffusion-weighted magnetic resonance imaging (DWI) in central retinal artery occlusion

	Optic nerve DWI +		Retina DWI +	
	Reader 1 (95% CI)	Reader 2 (95% CI)	Reader 1 (95% CI)	Reader 2 (95% CI)
Sensitivity	0.55 (0.32–0.76)	0.25 (0.10–0.49)	0.75 (0.51–0.90)	0.75 (0.51–0.90)
Specificity	0.70 (0.46–0.87)	1 (0.80–1)	0.80 (0.56–0.93)	1 (0.80–1)
PPV	0.65 (0.39–0.85)	1 (0.46–1)	0.79 (0.54–0.93)	1 (0.75–1)
NPV	0.61 (0.39–0.80)	0.57 (0.40–0.73)	0.76 (0.53–0.91)	0.80 (0.59–0.92)

*CI* confidence interval, *DWI* diffusion-weighted imaging, *NPV* negative predictive value, *PPV* positive predictive value

In comparison, DWI restrictions of the optic nerve were found in 11 (55%, reader 1) and 5 of 20 CRAO cases (25%; reader 2). Reader 1 falsely attributed optic nerve diffusion restrictions to 6 control patients (30%). Again, the assessment of reader 2 did not yield any false positives. Sensitivity, specificity, PPV and NPV for DWI restrictions of the optic nerve in CRAO were 55%/70%/65%/61% (reader 1) and 25%/100%/100%/57% (reader 2), respectively. Interrater reliability for optic nerve DWI restrictions was fair with unweighted Kappa κ = 0.32 (95% CI 0.09–0.56).

**Fig. 2:**
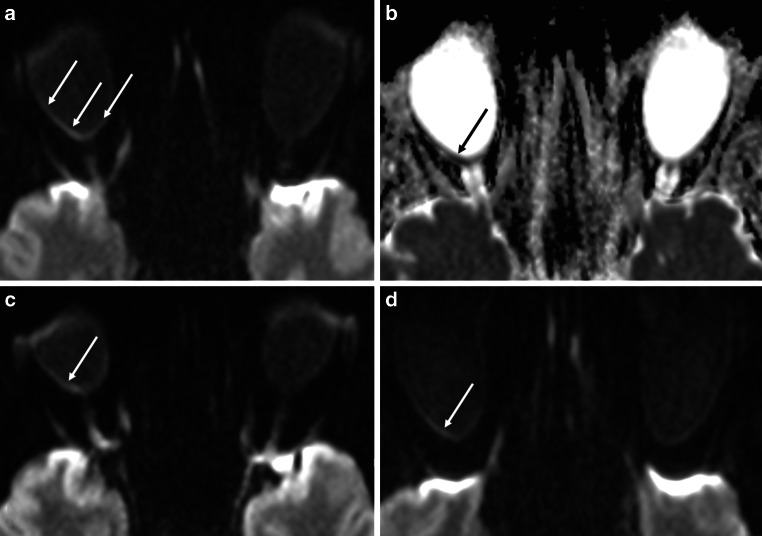
Retinal diffusion restrictions in acute right-sided central retinal artery occlusion in a 56-year-old patient with complete amaurosis. **a** On standard brain stroke DWI-EPI sequence (b = 1000 s/mm^2^) at 3 T the inner aspect of the posterior half of right globe appears clearly more hyperintense and is better delimitable as well as slightly thickened as compared to the contralateral side (*arrows*). **b** The ADC map image shows correspondingly reduced diffusion qualitatively (*arrow*). **c,** **d** DWI images above and below **a** showing the same image appearance with clearly positive DWI on the affected right side (*arrow*)

Representative examples of positive retinal DWI findings of CRAO are shown in Fig. 2. Figures of all unanimously positively rated cases can be viewed in the online supplement. Descriptively retinal findings included (1) a thin line of hypersignal along the inner aspect of the globe forming a U-shape and extending from the optic nerve head bilaterally up to or beyond the ora serrata, (2) a DWI hypersignal on either side of the optic nerve head nasally or temporally along the inner aspect of the globe, in most cases not extending to the ora serrata or (3) a mild regional hypersignal of the inner aspect of the globe with a mildly thickened depiction in comparison to the remaining parts of the globe.

## Discussion

In this preliminary retrospective case-control study retinal diffusion abnormalities can be seen in the majority of patients with CRAO on standard brain DWI. Diffusion abnormalities of the affected eye have been recently reported [26]; however, to the best of our knowledge, no systematic investigations have been performed. Using standard stroke DWI MRI at 1.5T or 3 T, we found retinal DWI restrictions in 75% of the CRAO patients from our cohort. Unweighted kappa indicated substantial interrater reliability (IRR) for retinal DWI restrictions. Both the reasonable sensitivity and specificity underline that retinal diffusion abnormalities seem to be a routinely applicable imaging finding that has been neglected so far. A potential reason might be the rather subtle nature of the findings on standard brain windowing, which become much more conspicuous after they have been called to one’s attention and are actively sought with appropriate windowing. Historically, the clinical application of ocular DWI is considered difficult because of the small size of the relevant structures, motion artifacts due to eye movement, high signal of the neighboring fat and fluid as well as image distortion secondary to magnetic susceptibility artefacts induced by neighboring bone and air [32]. With special respect to the retina, its delicate thickness of about 200 µm essentially made up by three serial neurons highly limits the ability to give clinically meaningful MR signal.

We found retinal DWI restrictions to be present between 17h and 105 h after onset of CRAO. Retinal DWI restriction dynamics in acute CRAO, however, remain elusive and should be a focus for future research. In our study, false negative assessments for retinal diffusion restrictions were exclusive to 1.5 T MRI. Increased field strength is therefore likely to improve sensitivity of retinal diffusion restrictions in CRAO. Further optimized retinal DWI protocols could include new sequence designs, such as non-EPI DWI sequences or readout-segmented multi-shot EPI techniques with motion correction and image reacquisition (i.e. RESOLVE technique, Siemens, Erlangen, Germany) as well as the use of additional orbital surface coils. These techniques have already been introduced into clinical practice and should be implementable with well-manageable efforts [33–37].

As diffusion restrictions of the optic nerve were only infrequently present in our collective of CRAO patients (sensitivity 25–55%) and unweighted kappa indicated only fair IRR, its diagnostic utility for diagnosis of CRAO seems limited. So far, only a few cases of diffusion restrictions of the optic nerve in CRAO have been reported in the literature [24, 25]. Bender et al. evaluated DWI in patients with acute loss of vision, including five patients with ischemic optic neuropathy (ION) [23]. All five patients showed diffusion restriction of the optic nerve; however, the ION etiology differed in all cases (hemorrhagic ischemia after gunshot trauma to the head, vasculitis in suspected giant cell arteritis, hypoxic brain damage and critical hypotension due to sepsis) and only one patient with iatrogenic embolic CRAO was evaluated. As diffusion restrictions of the optic nerve were also present in some patients with optic neuritis it is likely to lack specificity in the diagnosis of nonarteritic CRAO.

Our findings are limited by the retrospective design and small study size. Consequently, accuracy parameters need to be validated in future studies using extended cohorts. It is yet to be investigated whether retinal DWI restrictions are a specific finding to distinguish CRAO from other ophthalmologic causes of acute loss of vision. Furthermore, we did not perform ADC value measurements due to technical limitations (T2 shine through and partial volume averaging with resultant falsely high ADC values secondary to the extremely low thickness of the eyeball wall, the lack of an anatomically distinct retinal delineation and direct contact of the retina to high signal aqueous humour). In addition, time dependency of retinal DWI restrictions is not fully understood. The earliest positive DWI in our study was performed 17 h after symptom onset. Consequently, further studies are needed to confirm the presence of retinal DWI restrictions in hyperacute CRAO. Furthermore, we have not investigated potential T2-Fluid-attenuated inversion recovery (FLAIR) signal evolution of the restricted area as our T2-FLAIR sequences were acquired in the coronal plane and the slice thickness was 5 mm precluding any information on a potential extension of the DWI-FLAIR mismatch concept to CRAO for MRI-guided thrombolysis.

Our study presents the first systematic description and statistical evaluation of retinal DWI restrictions in CRAO. Apart from incidental descriptions in a single case report [27] and case series of 3 patients [26], no further studies of retinal DWI restrictions in CRAO currently exist. Our study design incorporated two blinded readers and a control cohort to analyze standard test quality criteria and interrater reliability of retinal DWI restrictions. As identical MRI protocols are used for CRAO and stroke/TIA patients in our department, cohorts were not discernible by differences in MRI sequence patterns. In addition, our control cohort prevented CRAO group identification through concurrent cerebral ischemia. Therefore, the assessment of retinal DWI restrictions with standard stroke DWI might provide supplementary evidence for the presence of CRAO in cases where ophthalmologic findings are uncertain.

The putative mechanism of the diffusion restriction on DWI likely parallels that of the brain tissue as retinal ganglionic cells are neurons and thus dependent on oxidative phosphorylation [38]. In fact, phylogenetically the retina belongs to the diencephalon [39]. In many cases we noticed a slight regional thickening of the eyeball wall on DWI, most likely secondary to codeveloping vasogenic edema, which appeared quite characteristic.

Studies in primates described extensive and irreversible retinal damage 105–240 min after selective clamping of the central retinal artery [40, 41]. Accordingly, investigations into the efficacy of intravenous or intra-arterial thrombolysis in CRAO may yield positive results if treatment is applied within a 4–4.5 h time frame [42–44]. As of now, randomized controlled trials of thrombolysis within 4.5 h of CRAO onset are still missing. This may in part be because the ophthalmological examination and cerebral imaging required usually delay time to treatment. Conceivably, retinal DWI may in the future be used to accelerate and secure the diagnosis of CRAO, especially in institutions where ophthalmological assessments are not routinely available. This may especially be relevant as accessibility of emergency brain MRI is likely to increase due to the recent advances in MRI-based treatment of ischemic stroke [45, 46].

To conclude, in this systematic study on DWI in acute CRAO, retinal abnormalities could be seen in the majority of patients on standard 1.5T and 3 T brain stroke DWI MRI not optimized to orbital imaging with good accuracy and IRR. Future studies will have to address pending issues, such as specificity and time dependency of imaging findings including the hyperacute phase, implement strategies to improve image quality and assess the clinical utility of retinal DWI for the differential diagnosis of acute vision loss and explore its potential value to guide recanalization treatment.

## Caption Electronic Supplementary Material

Supplemental methodological information.

Figure series of retinal diffusion restrictions in central retinal artery occlusion.
