# Supplementary material for: Standard Diffusion-weighted MRI for the Diagnosis of Central Retinal Artery Occlusion: A Case-Control Study
Source: Clin Neuroradiol. 2020 Sep 16;31(3):619–26. doi: 10.1007/s00062-020-00955-6 (PMC8463394; doi:10.1007/s00062-020-00955-6)
Supplement: Supplementary file 1 — Supplemental methodological information. [file 62_2020_955_MOESM1_ESM.docx]

Data Supplement

**Supplemental Methods**

**Vascular risk profile**

Cardiovascular risk factors were identified reviewing patient medical history, medication and laboratory test results. *Table 1* shows definition criteria of arterial hypertension, diabetes mellitus and dyslipidemia used in this study.

| Cardiovascular risk factor | Definition criteria |
| --- | --- |
| Arterial hypertension | Systolic blood pressure ≥ 140 mmHg, diastolic blood pressure ≥ 90 mmHg, treatment with antihypertensives and/or diagnosis of arterial hypertension in medical record |
| Diabetes mellitus | Glycated hemoglobin levels HbA_1c_ ≥ 6,5%/48 mmol/mol, antidiabetic treatment and/or diagnosis of diabetes mellitus in medical record |
| Dyslipidemia | LDL-cholesterol levels ≥ 130 mg/dl; HDL-cholesterol levels ≤ 35 mg/dl; statin treatment and/or diagnosis of dyslipidemia/hypercholesterolemia in medical record |

Table 1: Definition criteria of vascular risk factors.

**Ancillary diagnostics for CRAO etiology**

Etiologic classification of CRAO was based on the TOAST criteria[1]. Patients were screened for large artery atherosclerosis (LAA) using sonography (extracranial color-coded duplexsonography and transcranial color-coded-sonography TCCS), 3D time-of-flight magnetic resonance angiography and/or craniocervical computed tomography angiography. Etiology of CRAO was classified as “LAA” if patients presented >50% local stenosis (ECST ultrasound criteria for carotid stenosis[2]) of ipsilateral internal carotid artery or >50% stenosis (including occlusion) of major brain supplying artery with no indication of cardioembolism present.

12-lead electrocardiogram (ECG) and continuous ECG monitoring (24-72 hours) were reviewed for the presence of atrial fibrillation (AF). Transthoracic or transesophageal echocardiography identified medium- and/or high-risk sources of cardioembolism (CE) according to TOAST criteria[1]. Etiology of CRAO was classified as “CE” if patients (1) either exhibited or had a record of AF in medical history, and/or (2) medium or high-risk sources of cardioembolism was/were present in echocardiography and (3) large-artery atherosclerotic sources of CRAO were excluded.

We classified CRAO etiology as “undetermined” if no potential etiology of CRAO was found or two or more potential causes of CRAO were identified. Classification as “incomplete” was used in patients who did not receive echocardiographic evaluation and other ancillary diagnostics did not identify a potential CRAO etiology.

Sonography of the orbit was used to identify potential hyperechoic retinal artery embolus (“spot sign”), as described by Nedelmann et al.[3].

**Diffusion weighted MRI/Imaging Analysis**

MR imaging included an axial and sagittal T2 TSE, axial DWI, axial T2* or SWI, axial T1 SE or FLASH 2D, axial 3D TOF MRA sequences and a coronal T2-FLAIR sequence. Primary imaging modality were CT and CT angiography for all patients on admission or secondary on consultation by the neurologist in case of primary ophthalmological consultation. Imaging was performed on a 1,5T or a 3T scanner (Area and Skyra, respectively, both Siemens, Erlangen, Germany) with 32 channel head coils each. The routine stroke DWI sequences used had the following parameters: a) 3T: traced three-directional DWI EPI sequence, b-values 0 and 1000 s/mm2, slice thickness 3 mm, interslice gap 0.3 mm, number of averages 3, echo time 98, repetition time 10.3, number of phase encoding steps 143 , echo train length 71, percent sampling 100, percent phase field of view 100, pixel bandwith 1040, acquisition matrix 192, in-plane phase encoding direction AP, flip angle 90°, SAR 0.287, duration 2:36 min, b) 1,5T: traced three-directional DWI EPI sequence, b-values 0, 500 and 1000 s/mm2, slice thickness 3 mm, interslice gap 0.3 mm, number of averages 2, echo time 89, repetition time 8.80, number of phase encoding steps 105 , echo train length 53, percent sampling 80, percent phase field of view 100, pixel bandwith 1145, acquisition matrix 162, in-plane phase encoding direction AP, flip angle 90°, SAR 8.742, duration 2:22 min.

Images were evaluated as diagnostic or undiagnostic for orbital DWI image reading (severe artefacts obscuring at least one orbit were judged as non-diagnostic, frequently occurring image distortion was per se no reason for a categorization as non-diagnostic). Concerning the retina, several regional distributions of unilateral DWI signal elevation were regarded as positive: a thin line of hypersignal along the inner aspect of the globe forming a U-shape and extending from the optic nerve head bilaterally up to or beyond the ora serrata, a DWI hypersignal on either side of the optic nerve head nasally or temporally along the inner aspect of the globe, in most cases not extending to the ora serrata, or a mild regional hypersignal of the inner aspect of the globe with a mildly thickened depiction in comparison to the remaining parts of the globe.

The adapted Fazekas Score was evaluated as described previously to quantify leucoaraiosis[4]. Both periventricular changes and deep white matter changes were evaluated on a 4 point scale (0 = none to 3 = irregular and confluent lesions, respectively) separately and the higher subscale score was tabulated.

**Supplemental figures**


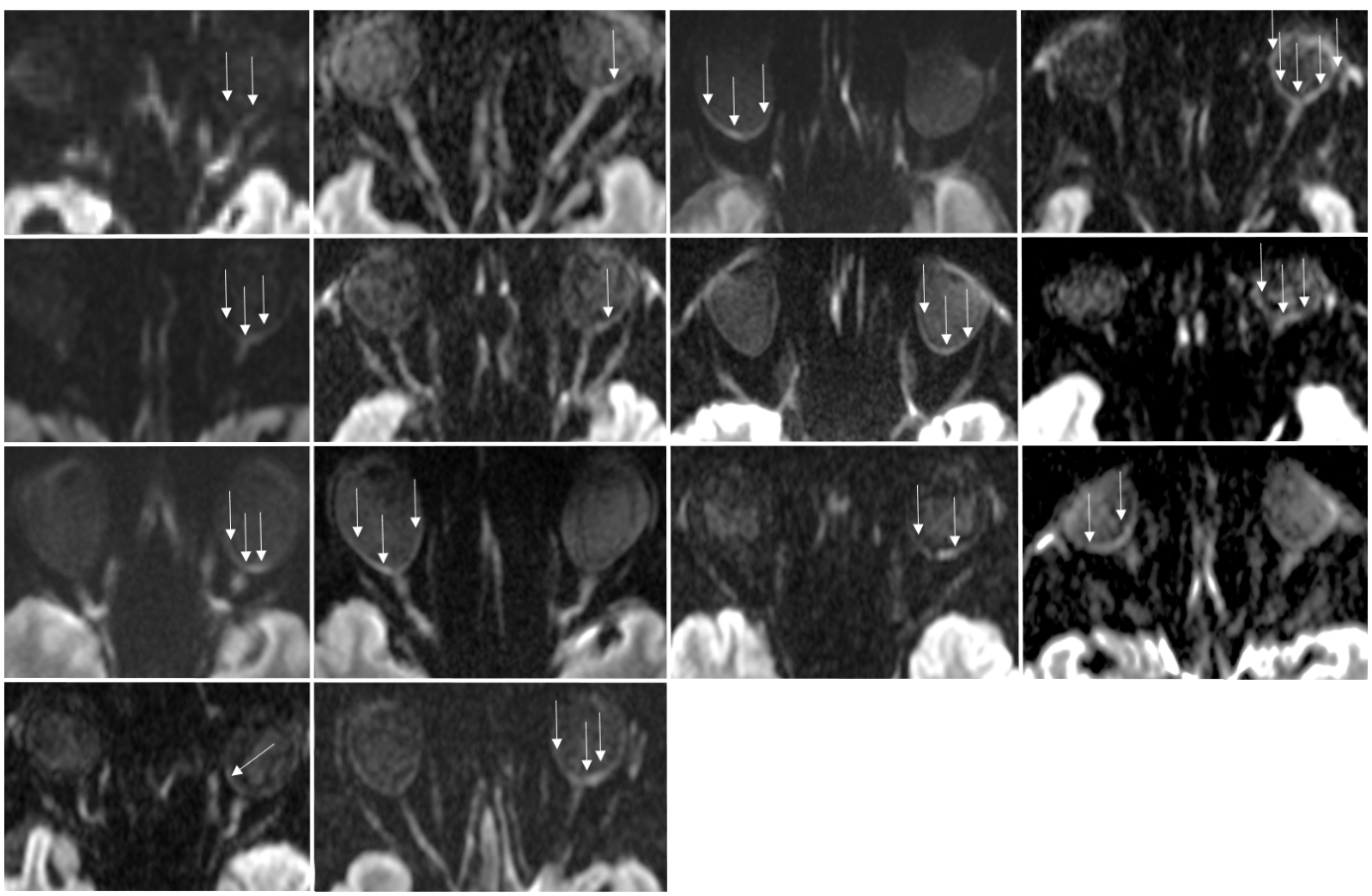


Figure 1: Standard brain stroke DWI-EPI sequences of 14 CRAO patients (at 1,5 or 3 Tesla). Retinal diffusion restrictions appear as a circumscribed hyperintense signal and thickening within the posterior half of the affected globe (arrows).

**References**

1. Adams HP, Jr., Bendixen BH, Kappelle LJ, Biller J, Love BB, Gordon DL et al. Classification of subtype of acute ischemic stroke. Definitions for use in a multicenter clinical trial. TOAST. Trial of Org 10172 in Acute Stroke Treatment. Stroke. 1993;24(1):35-41.

2. Randomised trial of endarterectomy for recently symptomatic carotid stenosis: final results of the MRC European Carotid Surgery Trial (ECST). Lancet. 1998;351(9113):1379-87.

3. Nedelmann M, Graef M, Weinand F, Wassill KH, Kaps M, Lorenz B et al. Retrobulbar Spot Sign Predicts Thrombolytic Treatment Effects and Etiology in Central Retinal Artery Occlusion. Stroke. 2015;46(8):2322-4. doi:10.1161/STROKEAHA.115.009839.

4. Thomalla G, Cheng B, Ebinger M, Hao Q, Tourdias T, Wu O et al. DWI-FLAIR mismatch for the identification of patients with acute ischaemic stroke within 4.5 h of symptom onset (PRE-FLAIR): a multicentre observational study. Lancet Neurol. 2011;10(11):978-86. doi:10.1016/S1474-4422(11)70192-2.
