# Supplementary figures and images for: Standard Diffusion-weighted MRI for the Diagnosis of Central Retinal Artery Occlusion: A Case-Control Study
Source: Clin Neuroradiol. 2020 Sep 16;31(3):619–26. doi: 10.1007/s00062-020-00955-6 (PMC8463394; doi:10.1007/s00062-020-00955-6)

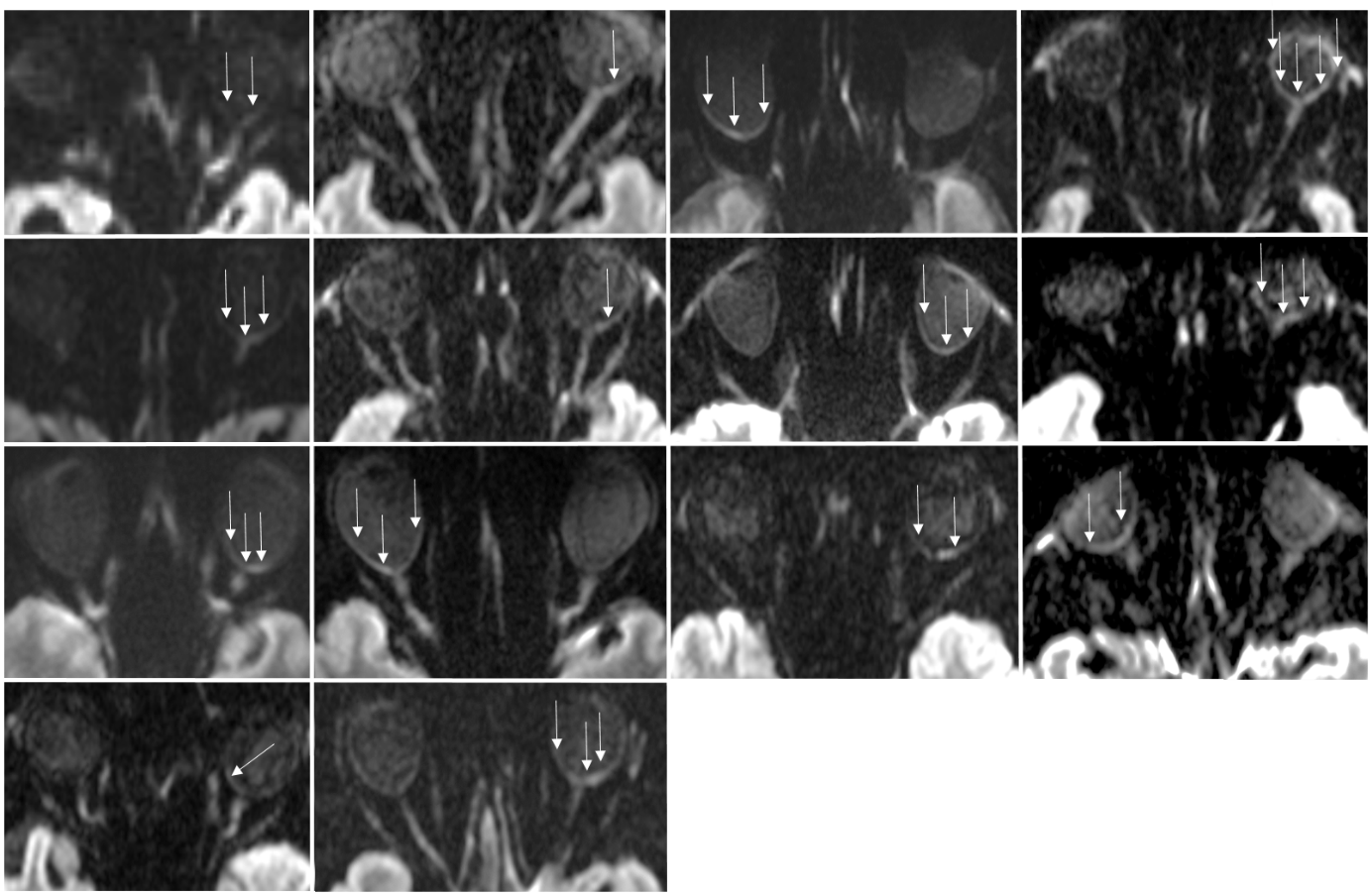

Supplement: Supplementary file 2 — Figure series of retinal diffusion restrictions in central retinal artery occlusion. [file 62_2020_955_MOESM2_ESM.tif]
